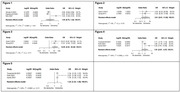# Association between number of teeth and cognitive decline: a systematic review and meta‐analysis of more than 4 million people

**DOI:** 10.1002/alz.091993

**Published:** 2025-01-09

**Authors:** Diego Chambergo‐Michilot, Rosa Montesinos, Belen Custodio, Eva Arias Rivera, Christopher Butler, Nilton Custodio

**Affiliations:** ^1^ Unit Cognitive Impairment and Dementia Prevention, Peruvian Institute of Neurosciences, Lima, Peru, Lima, Lima Peru; ^2^ Universidad Científica del Sur, Lima, Perú, Lima, Lima Peru; ^3^ Universidad Científica del Sur, Lima, Perú, Lima, Lima Peru; ^4^ Department of Brain Sciences, Imperial College London, UK, London, London United Kingdom

## Abstract

**Background:**

Dementia has sparked interest in identifying modifiable risk factors. It has been proposed that the number of teeth could play a crucial role in cognitive decline, suggesting possible mechanisms such as nutritional influence, inflammation, and neuronal feedback.

**Methods:**

We performed a systematic literature search in January 2024 in PubMed, Scopus and Embase to search observational studies that reported the association between number of teeth and dementia or cognitive decline. We pooled the reported or calculated odds ratios in forest plots.

**Results:**

We included nine studies in our systematic review. The total sample size was 4022654 patients. There was one study with more than 4 million people. Three studies reported the independent variable (number of present teeth) as a continuous variable, and six studies reported it as a categorical variable. Three studies reported the reference category as ≥ 20 present teeth, and the remaining three studies reported the reference category as ≥ 22 present teeth. The most common tool to define cognitive decline was the Mini‐Mental State Examination. We did not find a significant association between number of teeth as a continuous variable and dementia or cognitive decline (OR: 1.00, 95% CI: 0.70‐1.44) (Figure 1). Regarding the 3 studies with reference category ≥ 22 present teeth, having 11‐21 present teeth was not significantly associated with dementia or cognitive decline (OR: 2.61; 95% CI: 0.61‐11.06) (Figure 2), in contrast to people with 0‐10 present teeth where it was significantly associated with dementia or cognitive decline (OR: 14.58; 95% CI: 3.52‐60.40) (Figure 3). Additionally, the other 3 studies with reference category ≥ 20 present teeth, having 10‐19 present teeth was not significantly associated with dementia or cognitive decline (OR: 1.93; 95% CI: 0.87‐4.27) (Figure 4), however, having 1‐9 present teeth was significantly associated with dementia or cognitive decline (OR: 1.34; 95% CI: 1.32‐1.36) (Figure 5).

**Conclusion:**

A low number of teeth is associated with cognitive decline. Patients with cognitive decline should have a periodontal disease screening.